# Current Status and Future Directions of Ferroptosis Research in Breast Cancer: Bibliometric Analysis

**DOI:** 10.2196/66286

**Published:** 2025-02-26

**Authors:** Jia-Yuan Luo, Yu-Long Deng, Shang-Yi Lu, Si-Yan Chen, Rong-Quan He, Di-Yuan Qin, Bang-Teng Chi, Gang Chen, Xia Yang, Wei Peng

**Affiliations:** 1 Department of Pathology, The First Affiliated Hospital of Guangxi Medical University Nanning China; 2 Department of Oncology, The First Affiliated Hospital of Guangxi Medical University Nanning China; 3 Department of Hepatological and Gland Surgery, Wuzhou Gongren Hospital/The Seventh Affiliated Hospital of Guangxi Medical University Wuzhou China; 4 Day Chemotherapy Center, The First Affiliated Hospital of Guangxi Medical University Nanning China; 5 Department of Computer Science and Technology, School of Computer and Electronic Information, Guangxi University Nanning China

**Keywords:** breast cancer, ferroptosis, bibliometric, malignancy, cancer studies, treatment, bibliometric analysis, VOSviewer, China, United States, breast carcinoma, mammary cancer, strategy, trends, bibliography, review, disparities, forecast, treatment strategies, advancements

## Abstract

**Background:**

Ferroptosis, as a novel modality of cell death, holds significant potential in elucidating the pathogenesis and advancing therapeutic strategies for breast cancer.

**Objective:**

This study aims to comprehensively analyze current ferroptosis research and future trends, guiding breast cancer research advancements and innovative treatment strategies.

**Methods:**

This research used the R package Bibliometrix (Department of Economic and Statistical Sciences at the University of Naples Federico II), VOSviewer (Centre for Science and Technology Studies at Leiden University), and CiteSpace (Drexel University’s College of Information Science and Technology), to conduct a bibliometric analysis of 387 papers on breast cancer and ferroptosis from the Web of Science Core Collection. The analysis covers authors, institutions, journals, countries or regions, publication volumes, citations, and keywords.

**Results:**

The number of publications related to this field has surged annually, with China and the United States collaborating closely and leading in output. Sun Yat-sen University stands out among the institutions, while the journal *Frontiers in Oncology* and the author Efferth T contribute significantly to the field. Highly cited papers within the domain primarily focus on the induction of ferroptosis, protein regulation, and comparisons with other modes of cell death, providing a foundation for breast cancer treatment. Keyword analysis highlights the maturity of glutathione peroxidase 4-related research, with breast cancer subtypes emerging as motor themes and the tumor microenvironment, immunotherapy, and prognostic models identified as basic themes. Furthermore, the application of nanoparticles serves as an additional complement to the basic themes.

**Conclusions:**

The current research status in the field of ferroptosis and breast cancer primarily focuses on the exploration of relevant theoretical mechanisms, whereas future trends and mechanisms emphasize the investigation of therapeutic strategies, particularly the clinical application of immunotherapy related to the tumor microenvironment. Nanotherapy has demonstrated significant clinical potential in this domain. Future research directions should deepen the exploration in this field and accelerate the clinical translation of research findings to provide new insights and directions for the innovation and development of breast cancer treatment strategies.

## Introduction

Based on 2022 International Agency for Research on Cancer data, the global burden of breast cancer is alarming, with 2.3 million new cases, and over 665,000 deaths annually. Breast cancer constitutes 11.6% of new cancers and 6.9% of cancer deaths, underscoring the need for urgent research and intervention [1]. It has been projected that by 2040, due to global population growth and aging, the cancer burden will further increase, with breast cancer experiencing particularly notable growth. The number of new cases and deaths are expected to rise to over three million and one million, respectively [2]. As the most prevalent cancer worldwide, surpassing lung cancer, breast cancer poses a significant threat to human health. Despite the considerable progress achieved through sustained research in breast cancer diagnosis, treatment, and prevention, the intricate etiology of this malignancy remains widely undeciphered. Consequently, there is an urgent need to delve deeper into the complex mechanisms that underlie its development, with the aim of developing more precise and efficacious strategies for its management [3]. There is considerable variation in treatment response among individuals with this disease, and the prognosis and treatment outcomes for individuals diagnosed with advanced breast cancer are notably unfavorable [4]. Exploring new mechanisms can enhance our understanding of the biological behavior of breast cancer and improve treatment sensitivity.

Ferroptosis has added a new theoretical basis to the study of the molecular mechanisms underlying breast cancer. Since the introduction of this concept, the popularity of research in this field has rapidly increased, showing an exponential growth trend [5]. Ferroptosis is a recently discovered cell death mechanism mediated by iron-dependent phospholipid peroxidation and is tightly regulated by various cellular metabolic networks, including redox balance, iron handling, mitochondrial function, and amino acid, lipid, and glucose metabolism. This intricate regulation highlights its significance in breast cancer research [6]. Studies on breast cancer have revealed that the regulatory network of ferroptosis is sophisticated and complex, involving the synergistic effects of multiple signaling pathways and transcription factors. This regulatory network may modulate the malignant biological behavior of breast cancer cells via autophagy [7], metabolism [8], immune regulation [9], and other procedures, opening novel strategic directions for breast cancer treatment [5,6,10]. However, the literature concerning breast cancer and ferroptosis currently appears highly fragmented, and a systematic bibliometric analysis to consolidate this information is lacking, making it difficult to accurately summarize the research status and future direction of this field.

In response, this study aims to use bibliometric methods to conduct a comprehensive and objective analysis of the current status and future trends in breast cancer and ferroptosis research. This will entail a rigorous examination of pertinent indicators, including author distribution, research institutions, journals, country or region contributions, scientific publication output, highly cited papers, and keyword analysis. The ultimate objective of this study is to provide an in-depth revelation of the research hotspots and potential development trends in this field, thereby offering guidance for future research endeavors in exploring the mechanisms and therapeutic strategies related to ferroptosis in breast cancer.

## Methods

### Data Sources and Search Strategy

This study used data from two core collections within the Web of Science Core Collection (WOSCC): the Social Sciences Citation Index and the Science Citation Index Expanded. An advanced search was conducted using the search query (TS=(“Breast Tumor*” OR “Breast Cancer” OR “Mammary Cancer” OR “Breast Carcinoma” OR “Carcinomas, Breast”) AND TS = (“Ferroptosis”)), yielding a total of 624 papers. The search was further refined to include papers published between January 1, 2016, and February 1, 2024. Paper types were limited to original research papers, excluding reviews, conference abstracts, news items, advertisements, and non-English literature that did not meet the inclusion criteria. All data records in this study consisted of complete records and cited references and were exported in plain text format. To avoid errors and ambiguities due to database updates, all searches and data exports were completed on February 18, 2024. Additionally, all data in this study were obtained from open-source public databases; thus, no ethical issues were involved. The search and screening processes were independently conducted by JYL and YLD. In the case of any disagreements during the screening process, the final decision was reached after a discussion with the corresponding authors WP and XY.

### Inclusion and Exclusion Criteria

The inclusion criteria were (1) published journal papers; (2) papers with “breast cancer” and “ferroptosis” in the title, indicating a primary focus on breast cancer and ferroptosis; (3) papers not mentioning “ferroptosis” in the title but including ferroptosis-related research on breast cancer in the abstract; and (4) papers containing actual research on the relationship between breast cancer and ferroptosis within the full text.

The exclusion criteria were (1) nonliterature records, such as reviews, conference reports, briefings, news articles, editorial materials, letters, and advertisements; (2) non-English literature, (3) duplicate or data-deficient papers; and (4) withdrawn papers.

### Data Standardization and Visualization Methods in Bibliometrics

To ensure data accuracy and consistency, rigorous data standardization processing was applied to the raw data in this study. First, the study uniformly standardized the names of authors and institutions. Second, controversies over the attribution of country or region names were carefully resolved to ensure that the geographical attributes of the data were clear and unambiguous. In addition, synonymous keywords with the same meaning were merged, and keywords that were not directly related to the specific research content were deleted.

The study used the following tools for visualization and bibliometric analysis: the R package Bibliometrix (version 4.3.2; Department of Economic and Statistical Sciences at the University of Naples Federico II), VOSviewer (version 1.6.19; Centre for Science and Technology Studies [CWTS] at Leiden University), and CiteSpace (version 6.1.R6; Drexel University’s College of Information Science and Technology). The specific content included visual analyses of annual scientific publication output and citation trends, authors, institutions, journals, countries or regions, highly cited papers, and keywords. Notably, in VOSviewer, we applied “full counting” for normalized data processing. For cluster analysis of authors, institutions, and countries or regions, a minimum threshold of three publications and citations was set, and the clustering map of the top 100 keywords was further presented. Additionally, the Walktrap method was used in thematic map analysis for more precise topic delineation, while other analyses adhered to default software thresholds. By using these visualization methods, this study provided researchers in related fields with an intuitive and clear understanding of the research landscape.

### Ethical Considerations

No animal or human studies were carried out by the authors for this paper.

## Results

### Key Information

**Figure 1:**
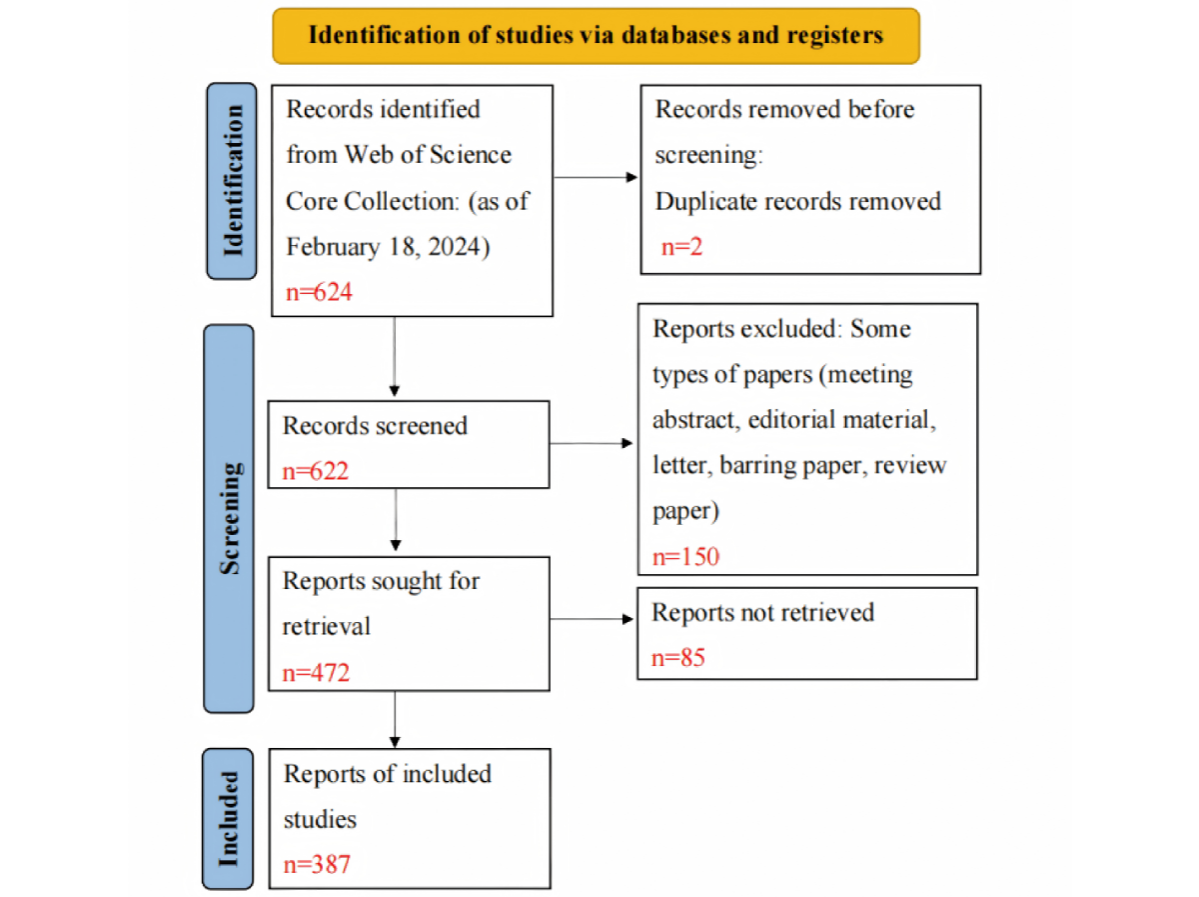
Flowchart of literature data included in this study.

This study included a total of 387 academic papers published by 2494 authors in 192 academic journals as the data source (the detailed data collection process is illustrated in Figure 1). On average, each paper had 8.26 coauthors, with only two papers authored by a single individual. The proportion of international collaborations was 16.54%. A total of 840 keywords and 15,107 cited references were identified. It is noteworthy that the average publication age of papers in this field was only 2.02 years, while the average number of citations per paper was as high as 27.22. These data demonstrate that this topic is rapidly emerging as a new research focus (Multimedia Appendix 1).

### Analysis of Scientific Output in Breast Cancer– and Ferroptosis-Related Research

#### Annual Analysis of Scientific Publications

**Figure 2:**
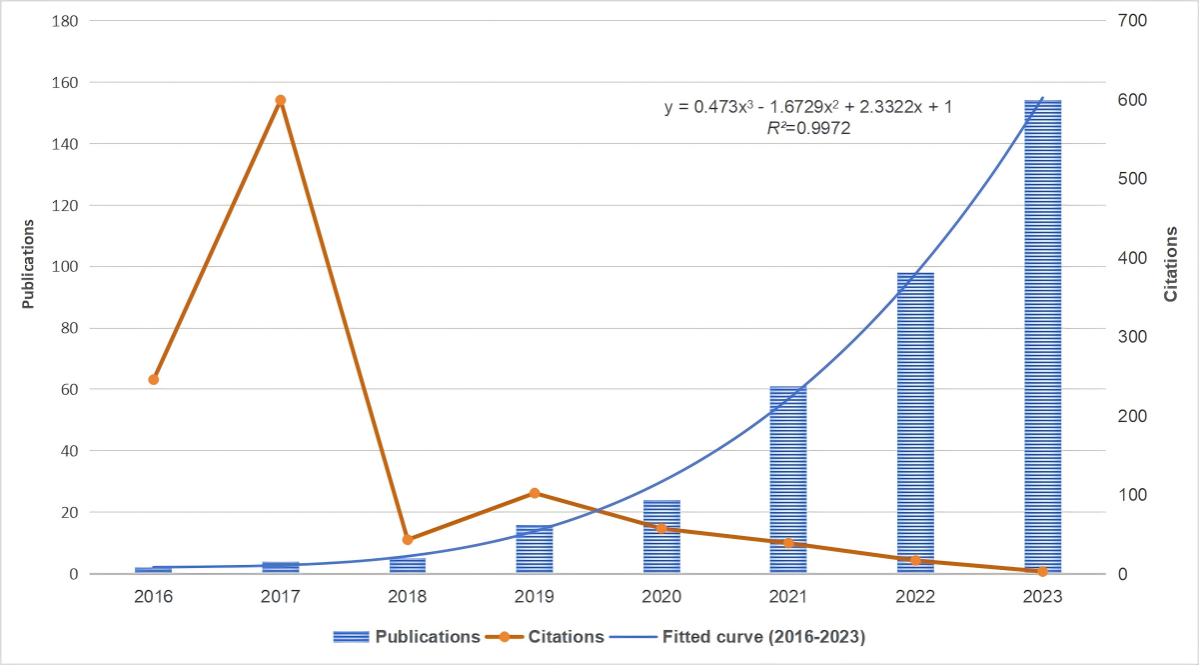
Visualization of annual publication and citation trends of papers (2016-2023). Note that the literature data for 2024 is only counted up to February 1, 2024, with 23 publications and 0 citations.

Due to the limited availability of relevant literature data published in 2024 up to the retrieval cutoff date of February 1, this study collected complete annual data spanning from 2016 to 2023 (n=364) from a total of 387 papers. A cubic function was fitted to the data to analyze the trend in publication volume (*R*²= 0.9972; Figure 2). Specifically, the bar chart indicates that the first paper in this field was published in 2016 (n=1), with 2023 marking the peak year of annual publications (n=154). The line graph represents the average annual citation frequency of relevant literature from 2016 to 2023, revealing a peak of 599 citations in 2017, followed by an overall decreasing trend in subsequent years.

#### Analysis of Scientific Publications by Prolific Authors

**Figure 3:**
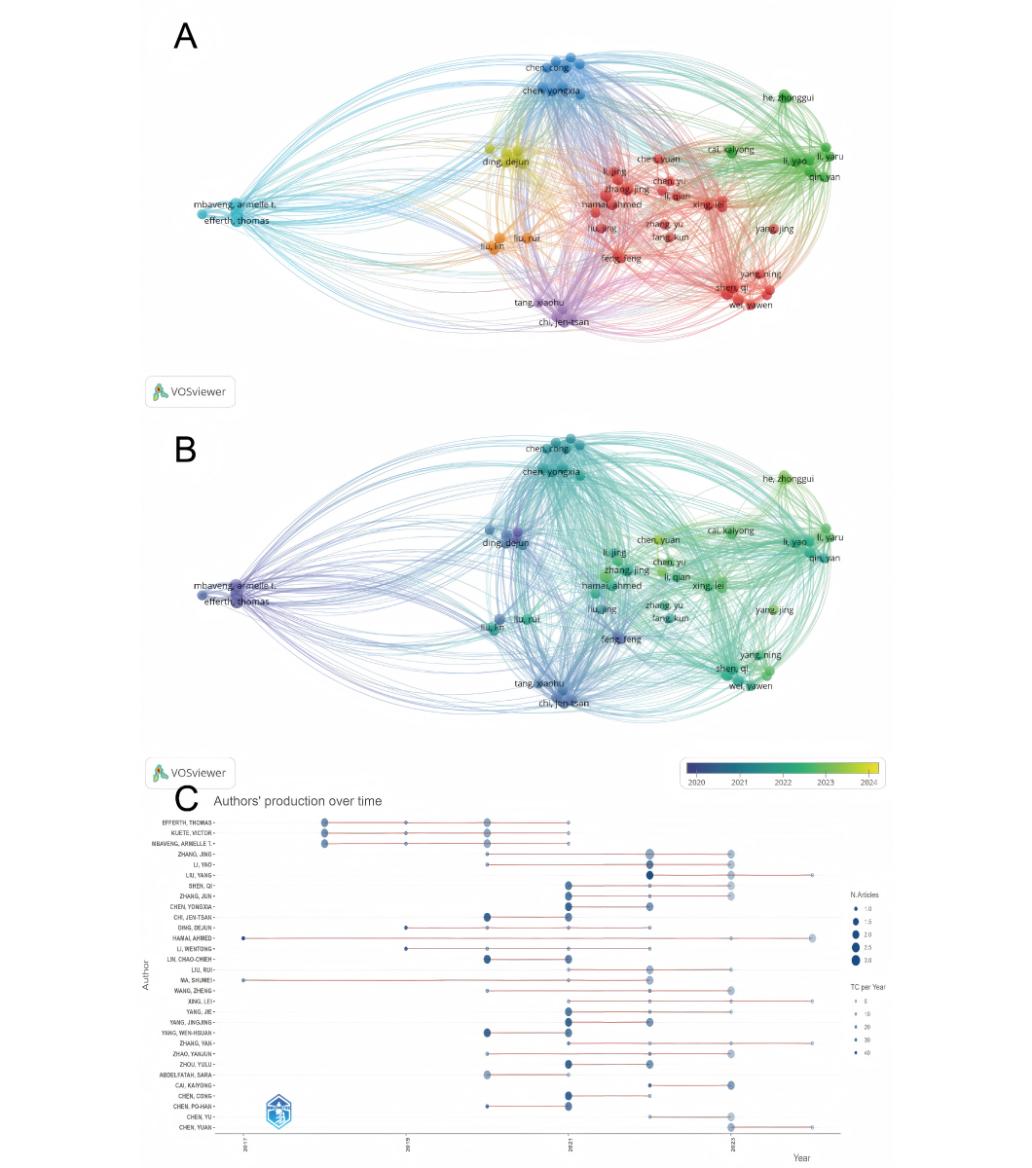
Visualization charts of authors’ publication volume and publication trends. (A) Network visualization map of authors’ publication volume. (B) Stacked visualization map of authors’ publication volume. (C) Heatmap of authors’ publication volume.

Through an analysis of the scientific publication output of prolific authors, the study found that 22 distinguished authors had published more than two papers in this research field, with no significant differences in the publication volume among the author groups in the field of ferroptosis and breast cancer. Notably, a minority of authors, represented by Efferth et al, had a significantly higher publication output, reaching six papers. Closely following them were authors from the Shen and Zhang groups, who have also contributed significantly to the lactoferrin research landscape, with a noteworthy publication count of five papers each, occupying important positions in the field of breast cancer and ferroptosis research (Figure 3A). It is noteworthy that, excluding the incomplete data from 2024, as shown in Figures 3B and 3C, an increasing number of new distinguished authors have emerged in recent years, with their publications mainly concentrated after 2020.

#### Analysis of Scientific Publications by Prolific Institutions

**Figure 4:**
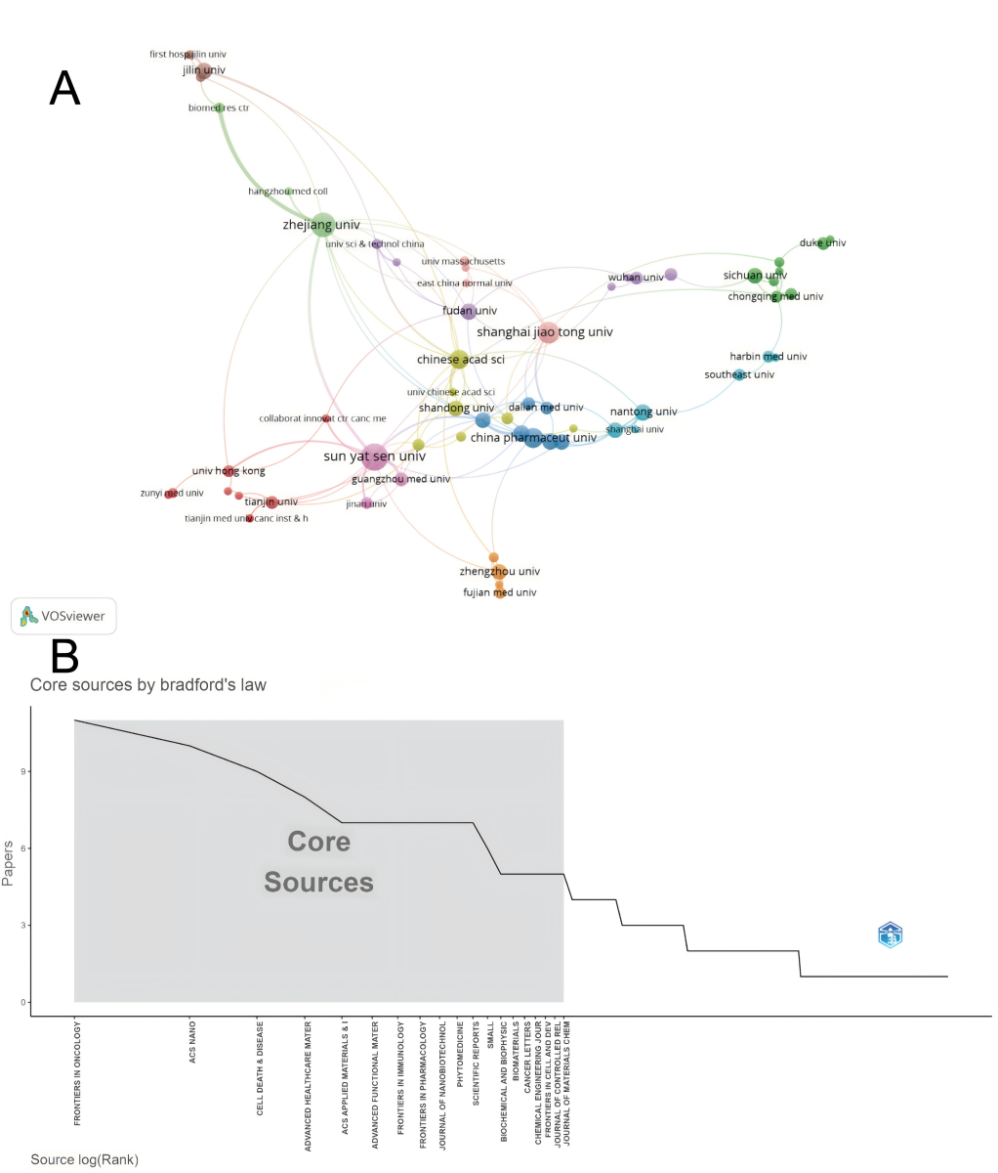
Visualization maps of institutions’ publication volume and identification of core journals. (A) Network visualization map of scientific institutions’ publication volume. (B) Identification of journals that have made significant contributions to the field, based on Bradford’s Law.

By analyzing the bibliometric data related to the research fields of breast cancer and ferroptosis, this study identified a group of institutions with significant scientific output in this area (Figure 4A). Among them, Sun Yat-sen University (n=23 publications) and Zhejiang University (n=19 publications) lead the list of institutions with publications related to breast cancer and ferroptosis. Apart from these two universities, other renowned domestic institutions, such as Shanghai Jiao Tong University (n=15 publications), China Pharmaceutical University (n=13 publications), and the Chinese Academy of Sciences (n=12 publications) have also performed exceptionally well in this field. It is also worth mentioning that non-Chinese institutions, such as Duke University (n=6 publications) and Stanford University (n=3 publications), also possess research capabilities in this area.

#### Analysis of Scientific Publications by Prolific Countries

**Figure 5:**
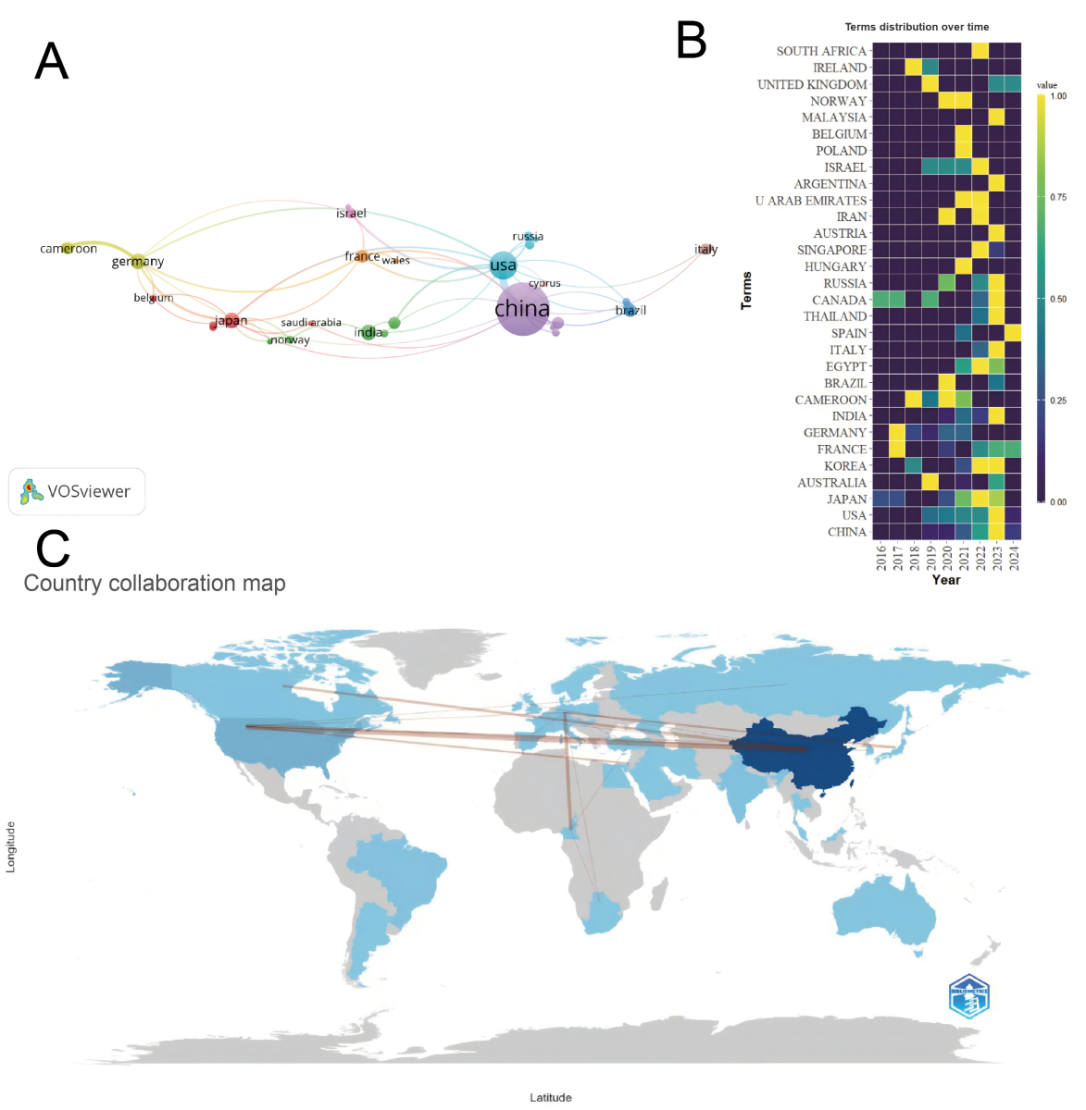
Global publication landscape and collaboration in breast cancer and ferroptosis research. (A) Network visualization map of country publication volume. (B) Heatmap of country publication trends. (C) Map of country collaboration relationships.

The study found that China (n=287) and the United States (n=52) occupy a dominant position in terms of paper output. Furthermore, countries such as Germany (n=11), India (n=11), Japan (n=11), France (n=7), Brazil (n=7), and Cameroon (n=6) have achieved certain research outcomes in the study of breast cancer and ferroptosis (Figure 5A). Furthermore, as depicted in Figure 5B, China experienced a pronounced acceleration in the volume of scientific publications focused on this research area around 2023, exhibiting a remarkable growth pattern. Moreover, the establishment of a close collaborative partnership between China and the United States emphasizes the inherently global nature of research endeavors in this domain (Figure 5C).

### Analysis of Citation Volume in Breast Cancer– and Ferroptosis-Related Research

#### Analysis of Frequently Cited Papers

As shown in the data presented in Multimedia Appendix 2, this study found that the highest citation count was from the paper “Ferroptosis is induced following siramesine and lapatinib treatment of breast cancer cells,” published in the journal *Cell Death & Disease*, with 38 citations. From the perspective of research focus and content, these citations primarily concentrate on the following aspects: first, the mechanisms and impacts of ferroptosis induction in breast cancer cells by distinctive drugs or therapeutic methods, as exemplified by papers 1, 2, 3, 4, 6, and 10; second, the exploration of the web-based mechanisms between related protein modulation and ferroptosis variations in breast cancer, as demonstrated in papers 2, 5, 7, 8, and 9; and third, the contrasts and interrelationships between ferroptosis and other modes of cell death in breast cancer cells, as discussed in paper 9.

#### Analysis of Frequently Cited Authors

The contributions and recognition of different authors can be reflected by the total citation count of their papers. According to the author citation analysis in Multimedia Appendix 3, Chen Y ranks at the top among all authors, with a total citation count of 64. Following closely is Gibson SB, with a total citation count of 51. Additionally, papers by Ding DJ, Li WT, Gai CC, Henson EE, Li ZHR, and Ma S have all been cited 38 times or more.

#### Analysis of Frequently Cited Countries

There are notable differences in the citations of research outcomes from different countries. According to the results shown in Multimedia Appendix 4, China ranks at the top in terms of total citations, with 4877, but the average number of citations per paper is 17.40. Conversely, Germany boasts the highest average citations per paper, reaching an impressive 489.20 citations. The United Kingdom, France, and Canada follow closely with average citations of 187.00, 118.70, and 108.80 per paper, respectively. It is noteworthy that the United States while ranking third after China and Germany in total citations with 1658, records an average of 50.20 citations per paper.

#### Overlay Analysis of High-Frequency Citing and Cited Journals

**Figure 6:**
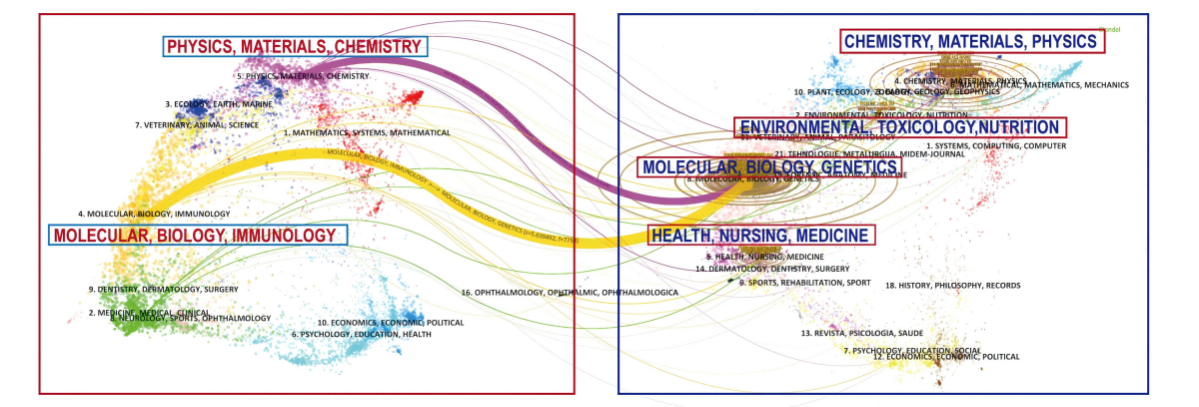
Dual-map overlay of journals illustrating the citation relationships between citing journals on the left and cited journals on the right. The width of the connecting lines represents the strength of the citation relationships.

In this study, CiteSpace software was used to construct a dual-layer overlay map of journals, visually representing the intricate relationships between citing journals (located on the left) and cited journals (located on the right) within the research domain of breast cancer and ferroptosis (Figure 6). The analysis results revealed that the group of citing journals represented by “Molecular, Biology, Immunology” and the core group of cited journals centered around “Molecular, Biology, Genetics” constitute the pivotal nodes in the knowledge flow path at the journal level (*z* score=5.639492; *f*=7758). Closely following this, there is a strong connection between the citing journal group of “Physics, Materials, Chemistry” and the cited journal group of “Molecular, Biology, Genetics” (*z* score= 2.1142766; *f*=3189).

### Cluster Analysis of High-Frequency Keywords and Main Evolution Directions in Breast Cancer– and Ferroptosis-Related Research

#### Cluster Analysis of Author Keywords and Analysis of Main Evolution Trends

After eliminating the core terms “breast cancer” and “ferroptosis,” along with their synonyms, this study conducted a clustering analysis of the top 100 most frequent author keywords. The results indicate that these keywords can be categorized into four primary clusters (Multimedia Appendix 5). Specifically, Cluster 1 (in red) predominantly focuses on research topics such as “triple-negative breast cancer” (n=56), “prognosis model” (n=18), and “cancer microenvironment” (n=17). Cluster 2 (in green) centers its research around concepts such as “peroxidation” (n=13), “photothermal therapy” (n=12), and the general term “cancer” (n=11). Cluster 3 (in blue) revolves around high-frequency keywords, such as “reactive oxygen species” (n=17), “gpx4” (n=14), and “stress” (n=13). Finally, Cluster 4 (in orange) concentrates on areas related to “cell death” (n=10), “anticancer” (n=6), and “drug delivery” (n=5).

**Figure 7:**
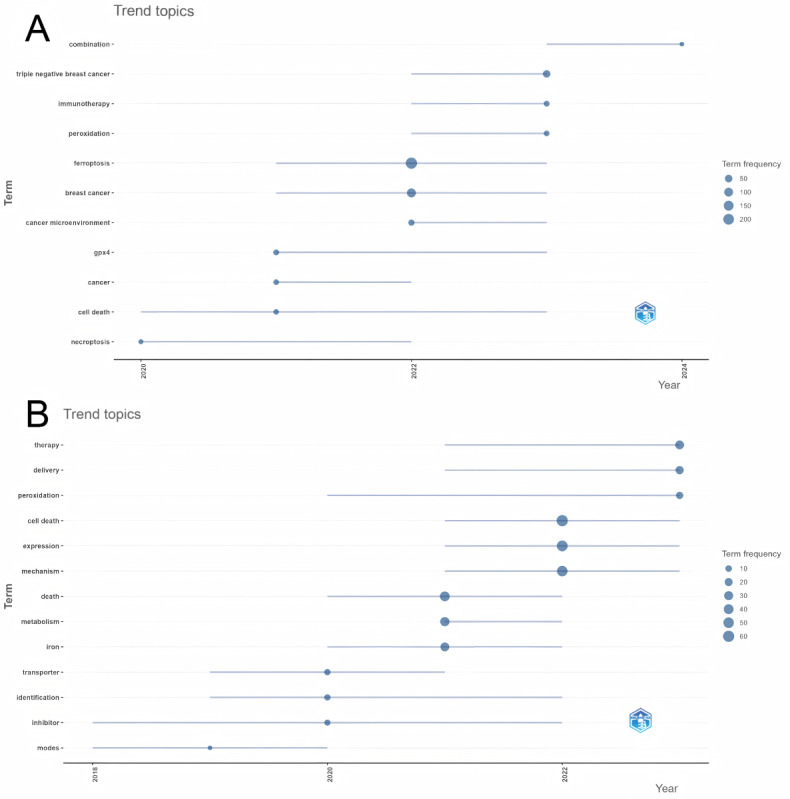
Trend charts of keywords. (A) Trend chart of author keywords. (B) Trend chart of Keywords Plus.

A deeper analysis was undertaken to examine the evolutionary patterns of high-frequency keywords over recent years, as presented in Multimedia Appendix 6A and Figure 7A. These trends reveal a progressive intensification of research focused on the concepts of cell death and glutathione peroxidase 4 (GPX4), indicating a growing interest and emphasis within the scientific community. It is noteworthy that from 2022 onwards, the frequency of keywords related to “cancer microenvironment,” “triple-negative breast cancer,” “immunotherapy,” “combination therapy,” and “peroxidation” increased significantly. Additionally, as witnessed from the time heatmap of the top 30 author keywords, keywords such as “erastin” and “autophagy” have maintained stable popularity in recent years (Multimedia Appendix 6; left panel).

**Figure 8:**
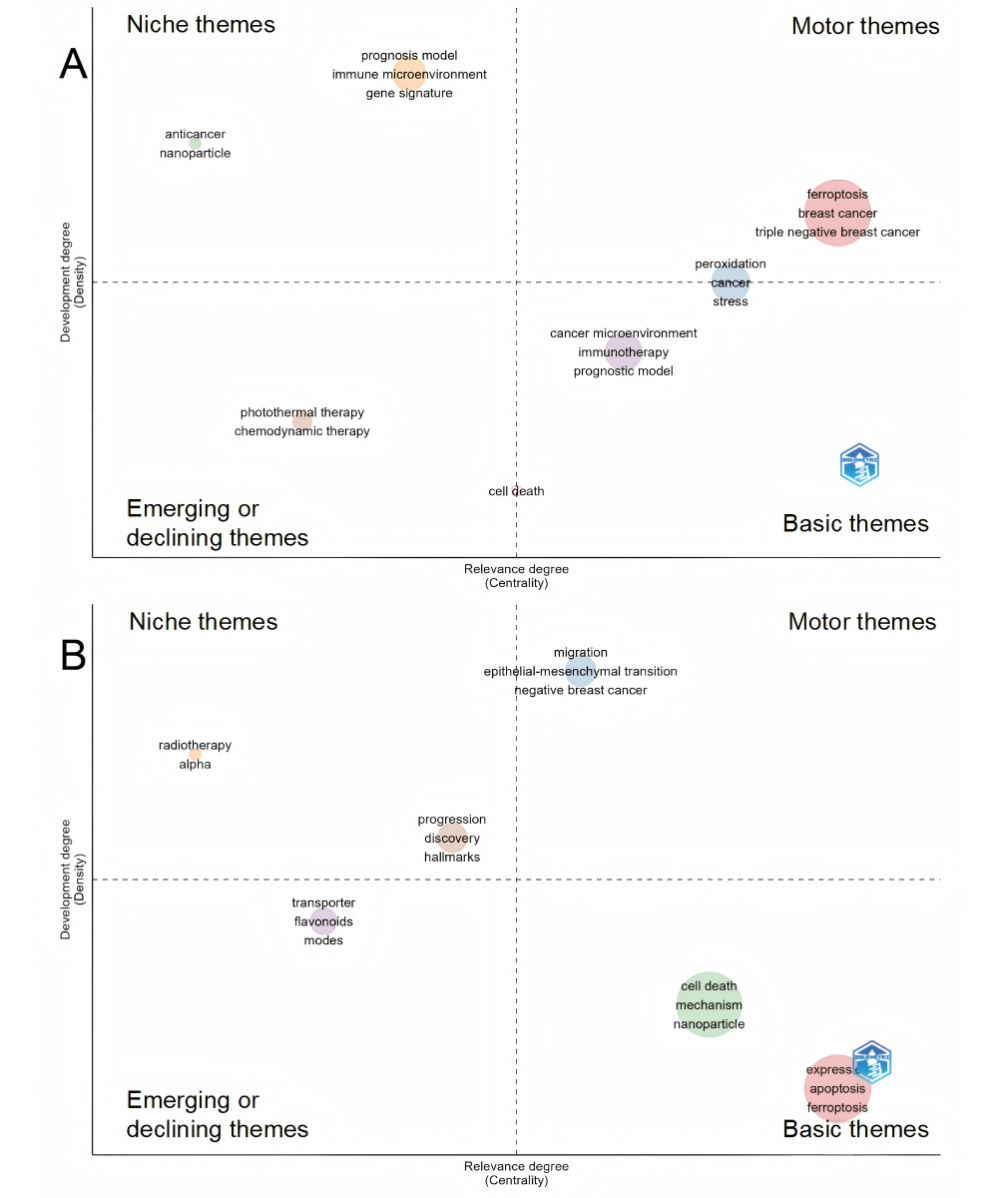
Thematic maps of keywords. (A) Thematic map of author keywords. (B) Thematic map of Keywords Plus.

Through a deep analysis of the thematic map data derived from the author keywords (Figure 8A), this study gained insights into the potential evolutionary directions in the research field of breast cancer and ferroptosis. The upper right quadrant of the map features motor themes, which incorporate the pivotal thematic terms including ferroptosis, breast cancer, and its subtype, triple-negative breast cancer (TNBC), underscoring their interconnectedness and centrality in the research landscape. The niche themes in the upper left corner encompass thematic keyword clusters such as anticancer and gene signatures. The emerging or declining themes in the lower left corner include topics such as chemodynamic therapy and photothermal therapy (PTT). Finally, in the lower right corner, the basic themes include thematic keywords such as cancer microenvironment, immunotherapy, and prognosis model.

#### Cluster Analysis of Keywords Plus and Analysis of the Main Evolution Trends in Research

The statistical data revealed that some papers had missing keywords, with an omission rate of 19.64%. By increasing the likelihood of yielding more precise outcomes, keywords derived using the Keywords Plus feature can enhance the rigor and reliability of research findings. Therefore, the following section presents a detailed analysis of Keywords Plus.

During the clustering analysis of Keywords Plus, this study excluded the core terms “breast cancer” and “ferroptosis,” along with their synonyms, focusing instead on the analysis of the top 100 most frequent terms. The analysis revealed that these terms could be grouped into five clusters (Multimedia Appendix 7). Among these clusters, the keyword “cell death” stands out as a focal point, with a high frequency of 99 occurrences. Additionally, “expression” (n=53), “mechanism” (n=49), “transporter” (n=19), and “stress” (n=9) were identified as core terms within their respective clusters.

This paper further analyses the evolution trends of keywords in the past and future. As shown in Multimedia Appendix 6 (right panel) and Figure 7B, the results indicate that researchers in the field of breast cancer and ferroptosis have consistently maintained a certain level of interest in the study of cell death in recent years. Additionally, newly emerged keywords such as “mechanism,” “expression,” and “nanoparticle” have been frequently observed in recent years.

Through the visualization presented in the thematic map (Figure 8B), the results indicate that the motor themes in the upper right quadrant consist of keywords such as “migration,” “epithelial-mesenchymal transition (EMT),” and “negative breast cancer.” The niche themes in the upper left quadrant encompass two core themes: “radiotherapy” and “progression.” Within the radiotherapy category, keywords such as “alpha” emerge, while in the progression category, keywords such as “discovery” and “hallmarks” appear. The emerging or declining themes in the lower left quadrant reveal keywords such as “transporter,” “flavonoids,” and “modes.” The basic themes in the lower right quadrant exhibit two main themes: “expression” and “cell death.” Within the expression category, keywords such as “apoptosis” and “ferroptosis” are present, while in the cell death category, keywords such as “mechanism” and “nanoparticle” emerge.

## Discussion

### Principal Findings

Breast cancer, as a malignancy with a high mortality rate, still faces significant limitations in early diagnosis and late-stage treatment. In recent years, ferroptosis, a unique form of cell death triggered by cellular metabolic imbalance, has attracted widespread attention [11], underscoring the importance and potential of research in this area. This study systematically reviewed 387 papers related to breast cancer and ferroptosis, retrieved from the WOSCC database, published between 2016 and February 1, 2024. The analysis revealed that the number of publications in this field peaked in 2023, showing a significant growth trend. However, the annual citation frequency of related publications exhibited fluctuations, with the highest citation rate observed in 2017, followed by a gradual decline. Further analysis highlighted the prominent contribution of scholars such as Efferth T, with Chen Y having the highest citation frequency. In terms of institutions, Sun Yat-sen University led in the number of publications. Research output from China and the United States dominated this field, with increasing collaborative efforts between the two countries. While China had the highest total citation count, Germany recorded the highest average citation frequency per paper. The journal *Frontiers in Oncology* published the highest number of papers. Citation network analysis indicated that journals in “Molecular, Biology, Genetics” were central to the citation network. High-impact papers focused on the induction mechanisms of ferroptosis in breast cancer and the regulatory role of related proteins. Keyword analysis identified GPX4 as a central research trend. Additionally, breast cancer subtypes emerged as key drivers of ferroptosis research. Fundamental research themes pointed to the tumor immune microenvironment, prognostic models, and nanotherapy as future research priorities.

### In-Depth Analysis of Publication Volume and Citation Counts in Breast Cancer and Ferroptosis Research

A comprehensive analysis of breast cancer and ferroptosis research reveals that publication volume and citation counts serve as crucial indicators for assessing the impact and development of this field. These metrics not only reflect the significance of research outcomes but also highlight emerging trends. An examination of frequently cited papers shows that key studies focus on the induction mechanisms of ferroptosis in breast cancer cells, protein regulation, and comparisons with other forms of cell death, aiming to discover novel therapeutic strategies. Specifically, the first study in this field published in 2016 demonstrated the effectiveness of siramesine and lapatinib in inducing ferroptosis in breast cancer cells [12], leading to high citation rates. Since then, exploration of the relationship between breast cancer and ferroptosis has deepened, with research becoming increasingly refined. TNBC, notable research by Yu et al [13] revealed that exosome-encapsulated erastin elevates ferritin levels, suggesting a new therapeutic approach. Hasegawa et al [14] further elucidated the intricate interplay between the cystine/glutamate antiporter, CD44v, and the MUC1-C oncoprotein in TNBC, enhancing our understanding of this subtype. Additionally, studies on the glutathione (GSH)-degrading enzyme CHAC1 have uncovered a novel mechanism of ferroptosis in TNBC via the GCN2-eIF2α axis [15].

The analysis of influential authors highlights those with high publication volumes and citation impacts. A distinguished team led by Efferth T has investigated the cytotoxicity of natural products, such as alkaloids and saponins, against multidrug-resistant cancers, focusing on their ability to induce various forms of cell death, including ferroptosis [16-21]. Their work provides important insights into overcoming drug resistance and developing novel anticancer therapies. High-impact authors, such as Chen Y and Gibson SB, have explored the therapeutic potential of ferroptosis in breast cancer, particularly through nanotechnology and drug design, contributing new perspectives to the field [22,23].

Institutional analysis reveals that Sun Yat-sen University and Zhejiang University lead in publication output in this area, reflecting their strong research foundations. Journals such as *Frontiers in Oncology*, *ACS Nano*, and *Cell Death & Disease* play pivotal roles in disseminating key findings and shaping the breast cancer and ferroptosis research landscape. By understanding the complex relationships between citing and cited journals, we uncover the distribution characteristics of core journals and knowledge flow paths within this interdisciplinary field, highlighting the contributions of journals from molecular biology, immunology, genetics, physics, materials science, and chemistry.

At the national level, China and the United States dominate in publications related to breast cancer and ferroptosis. China has made significant contributions to exploring the application of ferroptosis in cancer treatment, particularly through drugs, gene regulation, and nanotechnology. The United States, on the other hand, focuses on the mechanisms and regulation of ferroptosis in cancer, including its impact on lipid composition, iron metabolism, and key genes influencing ferroptosis sensitivity [23-27]. Despite limited resources, research teams from resource-poor countries like India and Brazil have leveraged nanotechnology to propose novel therapeutic strategies [28,29]. While China ranks first in total citations, Germany and the United States have higher citation rates per paper, indicating greater research impact and quality, possibly due to higher research investment and international collaboration.

The publication trend shows robust development in this field, with 28 publications by February 1, 2024, suggesting that 2024 may surpass 2023 in research output. The slight decline in citation rates reflects the surge in publications and underscores the growing interest in exploring the interplay between breast cancer and ferroptosis.

This growing research body highlights ferroptosis in breast cancer as an emerging frontier in biomedical science. Leading scholars in the field continue to make significant contributions, and institutions and countries demonstrate high levels of cooperation and investment. These positive factors predict that the field of breast cancer and ferroptosis research will achieve major breakthroughs in the near future, opening up new avenues for the diagnosis and treatment of breast cancer with profound implications.

### A Systematic Summary of Ferroptosis Mechanisms

Ferroptosis, characterized by its unique mechanism involving iron-dependent regulation through multiple pathways, plays a pivotal role in the onset and progression of various diseases. The core mechanism of ferroptosis lies in the imbalance between phospholipid peroxidation triggered by the oxidation of polyunsaturated fatty acids and the antioxidant system mediated by GPX4. When GPX4 activity decreases or GSH levels, as a cofactor, decline, phospholipid peroxidation products accumulate, leading to ferroptosis [5]. Methods to induce ferroptosis include the use of ferroptosis inducers (such as erastin and RSL3), inhibition of the system Xc-/GSH-GPX4 axis, and increased iron accumulation. Conversely, strategies to inhibit ferroptosis involve upregulating GPX4 expression, using ferroptosis inhibitors (such as ferrostatin-1), and enhancing the antioxidant system [7].

The signaling pathways and defense mechanisms of ferroptosis are complex and diverse. The initiation of iron toxicity involves multiple sources of reactive oxygen species (ROS), including iron-mediated Fenton reactions, mitochondrial ROS, and membrane-associated ROS driven by the NADPH oxidase (NOX) protein family. Cells defend against ferroptosis by upregulating antioxidant gene expression, repairing membrane damage, and clearing damaged organelles [30]. There is also a crosstalk between the mitochondrial dynamic regulatory network and ferroptosis, with mitochondrial dysfunction and damage potentially promoting ferroptosis [31]. By regulating lipid metabolism pathways, ferroptosis can affect cellular sensitivity to ferroptosis, thereby modulating its occurrence and development [32]. Furthermore, mechanisms such as epigenetic modifications and posttranslational modifications also play crucial roles in the regulation of ferroptosis [33].

Given that iron accumulation, oxidative stress, and lipid peroxidation are common features of various diseases, ferroptosis, as a unique cell death mechanism, may be a key link in the pathological processes of diverse diseases [34]. In neurodegenerative diseases, such as Parkinson disease and ischemic stroke, ferroptosis also plays a significant role [35]. In the field of cancer, ferroptosis is considered a potential antitumor strategy. For example, ferroptosis interacts with the tumor immune microenvironment, influencing tumor growth and metastasis [36]. Inhibiting ubiquitin-specific protease 8 can destabilize GPX4, thereby enhancing cancer cell sensitivity to ferroptosis, inhibiting tumor growth, and enhancing the efficacy of immunotherapy [37]. Additionally, ferroptosis is closely related to the death of plaque cells (such as vascular endothelial cells, macrophages, and vascular smooth muscle cells) and the development of atherosclerotic plaques in cardiovascular diseases [38]. Therefore, the application of ferroptosis in the treatment of various diseases, including cancer immunotherapy and neurodegenerative diseases, is gaining increasing attention.

Research on the regulatory mechanisms of ferroptosis in breast cancer indicates that its regulation involves multiple aspects, such as lipid metabolism and the immune microenvironment [39-42], and that ferroptosis plays a crucial role in the sensitivity and drug resistance of breast cancer to therapy [43-45]. With a deeper understanding of ferroptosis mechanisms, the methods for its detection are continuously evolving, including fluorescence probes, positron emission tomography imaging, and others, providing multiscale perspectives for ferroptosis research [46].

Future research should further elucidate the specific mechanisms of ferroptosis in the onset and progression of diseases, as well as its specific roles in the regulatory pathways, sensitivity, and resistance of breast cancer, providing new targets and strategies for breast cancer treatment.

### Keyword Analysis: Key Hotspot GPX4 and Ferroptosis Regulation in Breast Cancer

An analysis of evolutionary trends has revealed the increasingly prominent importance of GPX4 in relevant academic research fields. Previous studies have illustrated that GPX4, as a core regulatory factor of ferroptosis, plays a crucial role in organisms by catalyzing the reduction of phospholipid peroxides to inhibit iron ion degeneration, thereby affecting the ferroptosis process [47,48]. It has been reported that RUNX1 intron transcript 1 (RUNX1-it1) is significantly overexpressed in breast cancer tissues, and it can block the impact of ferroptosis by increasing GPX4 expression, thereby promoting the occurrence of breast cancer [49].

On the other hand, Tubastatin A, as an efficient and selective Histone Deacetylase 6 inhibitor, has been confirmed by relevant studies as a novel ferroptosis inducer. It promotes ferroptosis by inhibiting the activity of GPX4, thereby enhancing the effect of cancer radiotherapy [50]. Chloramine T has also been found to inhibit the cell growth of breast cancer and induce ferroptosis by targeting the GPX4 axis, indicating that it may become a potential new strategy for breast cancer treatment [51].

In addition, recent research has provided new insights into the regulatory mechanism of GPX4. As a crucial selenoprotein, the function of GPX4 in resisting ferroptosis depends greatly on the presence of its unique selenocysteine residue, which provides an important basis for understanding the mechanism of GPX4 [52]. The process of selenocysteine uptake mediated by low-density lipoprotein receptor-related protein 8 has been elucidated as a core mechanism in promoting GPX4 protein synthesis and effectively inhibiting ferroptosis [53,54].

Another study found that phospholipid-modifying enzymes MBOAT1 and MBOAT2, as ferroptosis inhibitors, regulate ferroptosis by remodeling the cellular phospholipid profile through the sex hormone signaling pathway [55]. These findings indicate that research on GPX4 and its related regulatory mechanisms is gradually deepening from phenotypic description to molecular mechanism analysis, offering unprecedented opportunities for the treatment of breast cancer.

### Motor Themes Analysis in Author Keywords: Differential Sensitivity of Ferroptosis in Various Breast Cancer Subtypes Driving the Field’s Development

Breast cancer is a highly heterogeneous disease, with different breast cancer cell types, characterized by the expression of estrogen receptor (ER), progesterone receptor (PR), human epidermal growth factor receptor 2 (HER2), and the proliferation marker Ki67, all exhibiting marked differences in sensitivity to ferroptosis. In the analysis of motor themes, findings related to ferroptosis sensitivity across breast cancer subtypes, including TNBC, have become well-established, closely interconnected with other emerging themes, and serve as a major driving force in the development of this field.

In ER-positive breast cancer, studies have shown that a combination of ferroptosis inducers, such as erastin and etoposide, can significantly enhance cellular sensitivity to ferroptosis by regulating IREB2/FPN1 expression and thus affecting iron metabolism, ultimately inducing ferroptosis [56]. Furthermore, ACSL4, a key regulator of ferroptosis, is upregulated in ER-positive breast cancer, promoting the accumulation of lipid peroxidation products and thereby enhancing cellular sensitivity to ferroptosis [57].

In contrast, sensitivity to ferroptosis in HER2-positive breast cancer may be regulated by multiple factors. For instance, inhibiting crVDAC3 can induce ferroptosis in breast cancer cells by reducing HSPB1 expression, thereby mediating resistance to deruxtecan in HER2-low breast cancer [58]. Additionally, m6A methylation modification of FGFR4 can inhibit ferroptosis, enhancing the resistance of HER2-positive breast cancer cells to anti-HER2 therapy [10]. Additionally, research has found that the inhibition of FGFR4 affects the expression of β-catenin/TCF4-SLC7A11 and FPN1, which has a significant impact on ferroptosis in HER2-positive breast cancer cells, thereby enhancing treatment sensitivity [10]. These studies suggest that the regulatory mechanisms of ferroptosis in HER2-positive breast cancer are more complex, involving the interaction of multiple signaling pathways.

In PR-positive breast cancer, although direct research on ferroptosis sensitivity is scarce, some indirect evidence suggests that PR may regulate the sensitivity of breast cancer cells to ferroptosis. Specifically, studies have reported that PR may indirectly regulate the sensitivity of TNBC to ferroptosis through metabolic pathways mediated by PR membrane component 1; however, these hypotheses await further experimental validation to establish their accuracy [59].

Relatively speaking, breast cancers that are negative for ER, PR, and HER2, especially TNBC, exhibit higher sensitivity to ferroptosis inducers. This characteristic may be closely related to the abnormal disruptions in iron and lipid metabolism in TNBC, which make TNBC cells more susceptible to ferroptosis inducers [60,61]. For example, the high expression level of ACSL4 in TNBC promotes the generation of lipid peroxides, a core step in the execution of ferroptosis [62]. Studies have also found that ferroptosis is enhanced through the combined action of GSH depletion and dihydroorotate dehydrogenase inhibitors [61]. Furthermore, the functions of key regulatory factors, such as the Xc-/GSH/GPX4 axis, ACSL4/LPCAT3 pathway, and nuclear factor erythroid 2-related factor 2 (NRF2) in ferroptosis, as well as their potential dysregulation mechanisms related to cancer cell survival and drug resistance, have been thoroughly explored [60]. Additionally, some natural compounds, such as artemisinin derivatives and traditional Chinese medicine extracts, have been found to inhibit the growth of TNBC cells by inducing ferroptosis [63].

Ki67, a commonly used proliferation marker, is often associated with higher proliferative activity and malignancy in breast cancer. Interestingly, breast cancer cells with high Ki67 expression also exhibit higher sensitivity to ferroptosis inducers [64]. However, research on the direct relationship between Ki67 expression and ferroptosis sensitivity is still limited. Future studies should further explore the role of Ki67 in the regulation of ferroptosis and whether it can serve as a biomarker for predicting the therapeutic effect of ferroptosis induction.

The motor themes analysis has provided a solid foundation for research in the area of breast cancer and ferroptosis. Continued investigation into the interactions between differential ferroptosis sensitivity across breast cancer subtypes holds the potential to uncover new therapeutic targets and biomarkers, ultimately driving the development of precision medicine and improving treatment outcomes and prognoses for patients with different breast cancer subtypes.

### Analysis of Basic Themes in Breast Cancer and Ferroptosis Research: Immune Microenvironment and Prognostic Models Reveals Future Directions

The basic themes of immune microenvironment and prognostic models in breast cancer and ferroptosis research are closely interrelated and have led to several significant findings. For instance, in an insightful review by Shen et al [65], the authors explicitly point out the central role of iron in innate and adaptive immune responses, further emphasizing the potential positive influence of targeted regulation of iron metabolism on antitumor immunity and cancer treatment. Recent studies have demonstrated that T-cell–mediated immune responses [65] and short-term acidosis-induced M1 macrophage polarization can effectively promote ferroptosis [66], thereby playing an active role in antitumor immunotherapy.

Research undertaken by Xu et al [67] has elegantly demonstrated that the development of ferroptosis-related gene (FRG) signatures holds significant promise as robust prognostic indicators for the immune microenvironment and therapeutic responsiveness. This approach shows great potential to inform and guide the design of future individualized treatment strategies, thereby enhancing precision medicine in breast cancer management.

A new lncRNA signal closely related to ferroptosis has been discovered, with its immune-related pathways significantly enriched in high-risk patients with breast cancer, demonstrating great potential for predicting the prognosis of patients with breast cancer [68]. Knocking down the hub gene SLC39A7, combined with TME scoring, can significantly affect the apoptosis and ferroptosis of cancer cells, with higher prognostic efficacy.

Meanwhile, epithelial cells and B cells exhibit higher ferroptosis scores, which are respectively related to the immune checkpoint blockade response and immune checkpoint blockade-independent gene expression, suggesting a connection between ferroptosis and the immune microenvironment [69]. It is noteworthy that research has accurately predicted the survival of patients with breast cancer through a novel prognostic model composed of nine FRGs [70].

Chen et al [71] used the Cancer Genome Atlas to identify 11 ferroptosis genes related to breast cancer prognosis, established a precise prognostic model, and analyzed therapeutic targets, thereby enhancing prognosis management. In TNBC, the subtype of breast cancer with the worst prognosis, research by Wu et al [72] evaluated the role of FRGs and found that they may affect prognostic models by regulating the tumor microenvironment, thus providing a solid theoretical basis for a clinically accurate prediction of TNBC prognosis. Furthermore, studies have evaluated the FRG AKR1C1, revealing its potential related to the immune-microenvironment, which may further affect the progress and prognosis of patients with breast cancer, making it a novel biomarker for the immune microenvironment and prognosis determination of breast cancer [73].

In conclusion, although significant progress has been made in the areas of immune microenvironment and prognostic models within the context of breast cancer and ferroptosis research, these fields remain underexplored and offer considerable potential for future investigation. These themes provide valuable directions for further research and offer essential guidance for researchers aiming to deepen their work in this area.

### Innovative Applications and Strategies of Nanoparticles: Further Supplementing Future Research Directions in Breast Cancer and Ferroptosis

Through an in-depth analysis of the Keywords Plus basic themes, we further explore the future development directions of nanoparticles in the context of breast cancer and ferroptosis research. The application of nanoparticles in breast cancer treatment relies heavily on their unique physical and chemical properties to achieve the targeted delivery and controlled release of drugs, thereby enhancing therapeutic efficacy and reducing side effects [74]. Nanoparticles can exert their effects through various mechanisms that promote cell death, including ferroptosis [75]. Recent research has reported the use of a novel Fenton-independent pathway using photothermal nanozymes to overcome the limitations of the Fenton reaction. By using iron-containing hollow mesoporous Prussian blue (HMPB) nanocubes as an iron source to prepare HMPB@Lip, which combines PTT with nanozyme action, the induced ferroptosis through a non-Fenton reaction effectively ablates tumors, demonstrating the therapeutic potential of HMPB@Lip as a multifunctional nanozyme for ferroptosis. Furthermore, the development of Her2-DSG NPs nanostructures, which combine chemo-PTT with tumor microenvironment remodeling and immune activation, holds great promise for HER2-positive breast cancer [76].

Another study constructed nFeAPG nano-complex synergizing Fe³⁺ with apigenin to enhance immune response through PTT, effectively controlling TNBC [71]. Additionally, the ICG@SANPs-cRGD nanomaterial-mediated combination of PTT/photodynamic therapy and immunotherapy has demonstrated its potential as a multifunctional platform for breast cancer treatment [77].

Furthermore, a search conducted on the Clinical Trials website revealed that there is only one clinical trial project related to breast cancer and ferroptosis, and that it is associated with nanocarriers (Multimedia Appendix 8). As of the search date, November 12, 2024, this project is still in the recruiting phase. Its focus is on the application of carbon nanoparticle-loaded iron (CNSI-Fe (II)) in the treatment of patients with advanced solid tumors, particularly those who have experienced treatment failure (including disease progression or intolerance) or lack of available standard therapeutic options (trial identifier: NCT06048367). The primary end point of this study is to assess the safety and tolerability of CNSI-Fe (II) throughout the study duration. The secondary end point, meanwhile, focuses on evaluating the pharmacokinetic profile of CNSI-Fe (II) in patients with advanced solid tumors.

Nanotechnology, as a fundamental topic in the research fields of breast cancer and ferroptosis, is still in its relatively early stages of development. Nonetheless, these research achievements have not only significantly expanded the application scope of nanotechnology in breast cancer treatment but have also highlighted its close connection with breast cancer therapy and identified numerous promising therapeutic research directions that await further exploration. More importantly, they have pointed out new avenues for future research endeavors and provided invaluable guidance for investigative pursuits.

### Limitations

This study represents the first comprehensive bibliometric analysis of 387 academic papers related to breast cancer and ferroptosis sourced from the WOSCC database. By applying multiple analytical approaches, it thoroughly investigates the research hotspots and future trends in this field, offering a comprehensive and systematic overview of the latest developments in this area. These strengths significantly enhance the academic value and practical relevance of the study, providing essential guidance for future advancements in the field of breast cancer and ferroptosis.

However, several limitations of our methodology must be acknowledged. First, although the WOSCC database is vast, it was not specifically designed for bibliometric analysis and relies on a single data source, which may not capture all relevant literature present in other databases (such as PubMed, Scopus, and Google Scholar), potentially introducing bias. Future research should integrate multiple databases to mitigate this issue. Second, our focus on English-language publications limits the comprehensiveness of the study by excluding high-quality non-English research. To address this, future studies should adopt multilingual search strategies. Third, the exclusion of nonpaper publications (eg, reviews and conference papers) constrains the scope of research outputs considered. To obtain a more holistic view, future work should adopt more inclusive selection criteria. Finally, given the rapid updates in databases, there is a risk of overlooking recent advances in the field’s literature. Periodic updates to bibliometric analyses are recommended to ensure timely and accurate capture of the latest trends and developments in breast cancer and ferroptosis research.

### Conclusions

Ferroptosis holds significant value and potential in breast cancer research. Through bibliometric analysis, this study suggests that enhancing collaboration and communication among authors, institutions, and countries, while leveraging the interdisciplinary nature of journal citations, can facilitate advances in fundamental research on ferroptosis in breast cancer. Specifically, this paper reviews the progress in understanding the mechanisms of ferroptosis, providing guidance for breast cancer research. It emphasizes the sensitivity of different breast cancer subtypes to ferroptosis as a driving force in the current research landscape and highlights the pivotal role of GPX4 in the regulatory mechanisms of ferroptosis in breast cancer as a key focus. Additionally, the study identifies the importance of the tumor microenvironment, immunotherapy, and prognostic models for future development. Although the application of nanoparticles in breast cancer treatment is in its preliminary stages, it has already shown promising prospects and serves as a rich supplement to the future directions. By elucidating the current status and future trends of ferroptosis in breast cancer research, this study provides direction for future research endeavors and promotes innovation in breast cancer treatment strategies.

## Appendix

Multimedia Appendix 1 Key information of the included studies.

Multimedia Appendix 2 Top 10 most frequently cited articles in research on ferroptosis and breast cancer.

Multimedia Appendix 3 Top 10 authors with the most citations in ferroptosis and breast cancer research within the field.

Multimedia Appendix 4 Top 10 countries ranked by total citations and average article citations in the field of ferroptosis and breast cancer research.

Multimedia Appendix 5 Author keywords clustering visualisation in the field of breast cancer and ferroptosis research.

Multimedia Appendix 6 Heatmap of keyword analysis.

Multimedia Appendix 7 Visualisation of keywords plus clustering in breast cancer and ferroptosis research fields.

Multimedia Appendix 8 Summary of clinical trials in the field of ferroptosis and breast cancer research (n=1).

## Abbreviations

ER: estrogen receptor
FRG: ferroptosis-related gene
GPX4: glutathione peroxidase 4
GSH: glutathione
HER2: human epidermal growth factor receptor 2
HMPB: hollow mesoporous Prussian blue
PPT: photothermal therapy
PR: progesterone receptor
ROS: reactive oxygen species
TNBC: triple-negative breast cancer
WOSCC: Web of Science Core Collection

## Data Availability

The datasets generated during or analyzed during this study are available from the corresponding author upon reasonable request.
