# Supplementary figures and images for: Current Status and Future Directions of Ferroptosis Research in Breast Cancer: Bibliometric Analysis
Source: Interact J Med Res. 2025 Feb 26;14:e66286. doi: 10.2196/66286 (PMC11904379; doi:10.2196/66286)

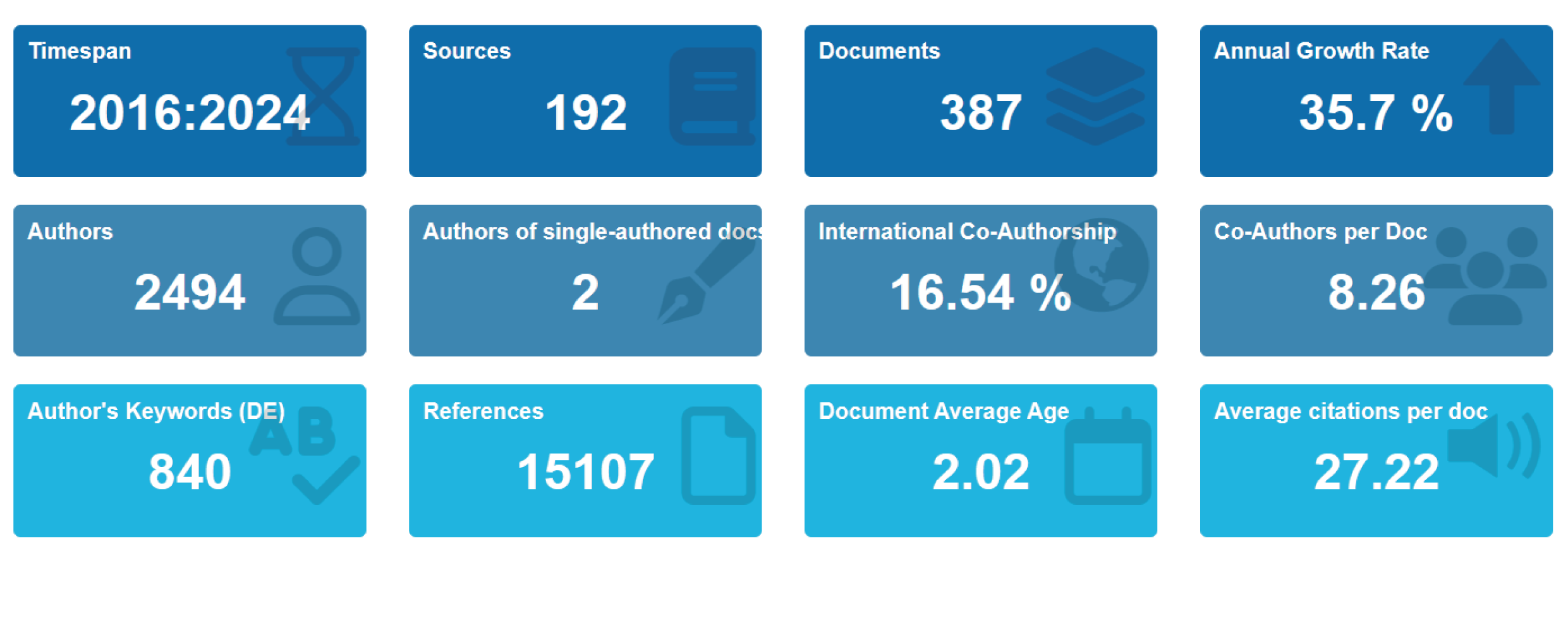

Supplement: Multimedia Appendix 1 [file ijmr_v14i1e66286_app1.png]

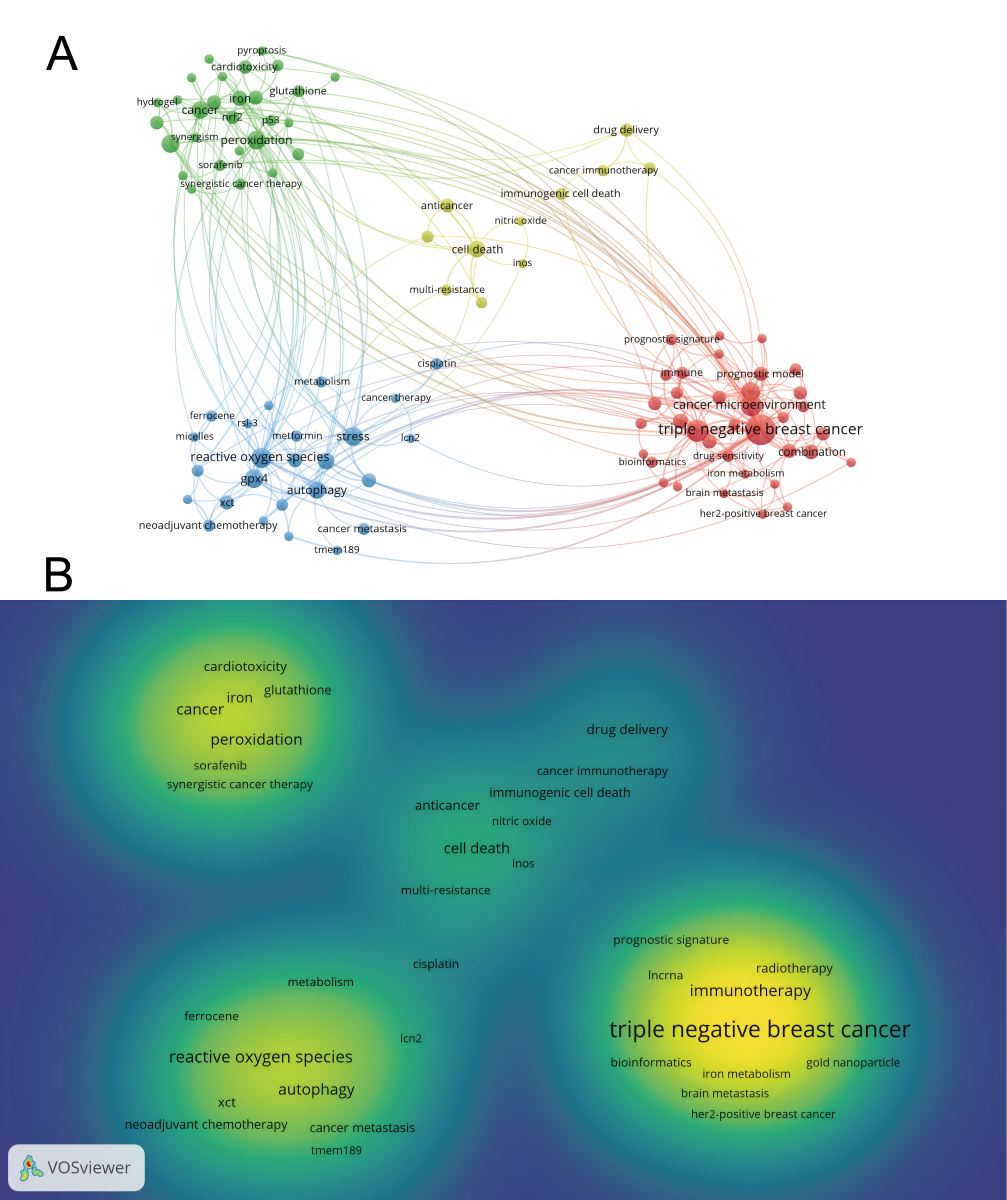

Supplement: Multimedia Appendix 5 [file ijmr_v14i1e66286_app5.png]

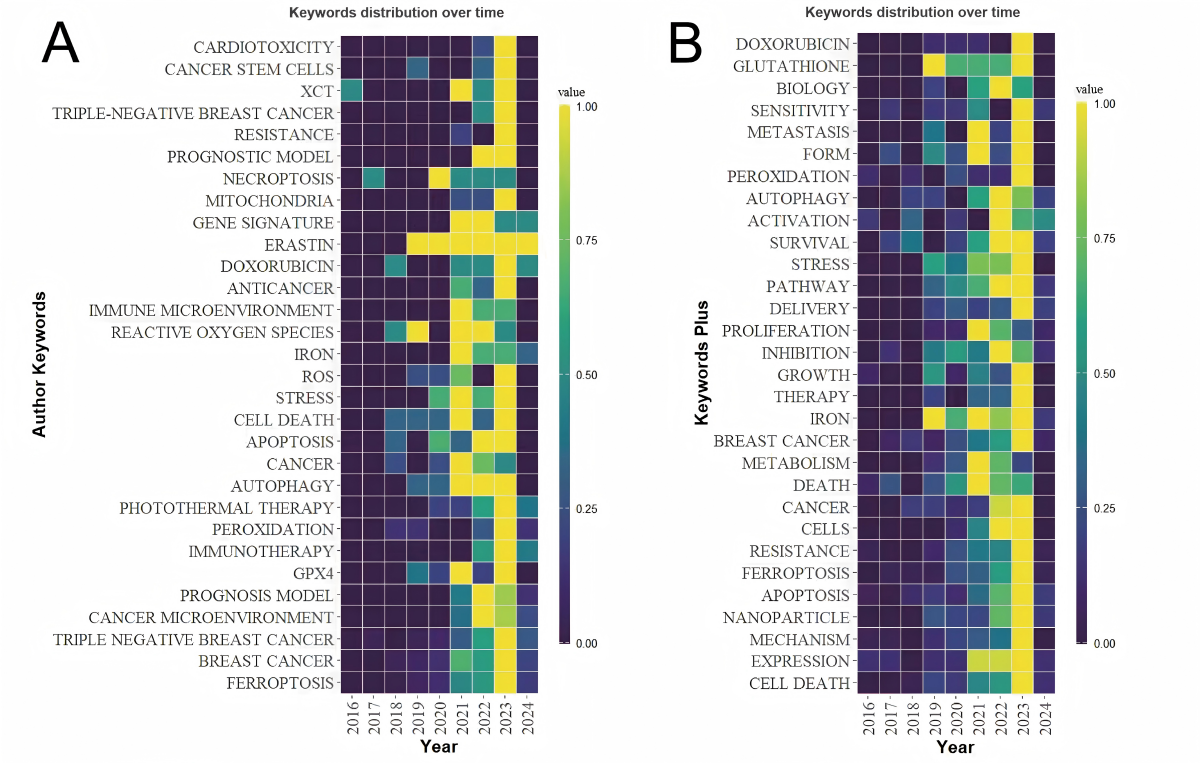

Supplement: Multimedia Appendix 6 [file ijmr_v14i1e66286_app6.png]

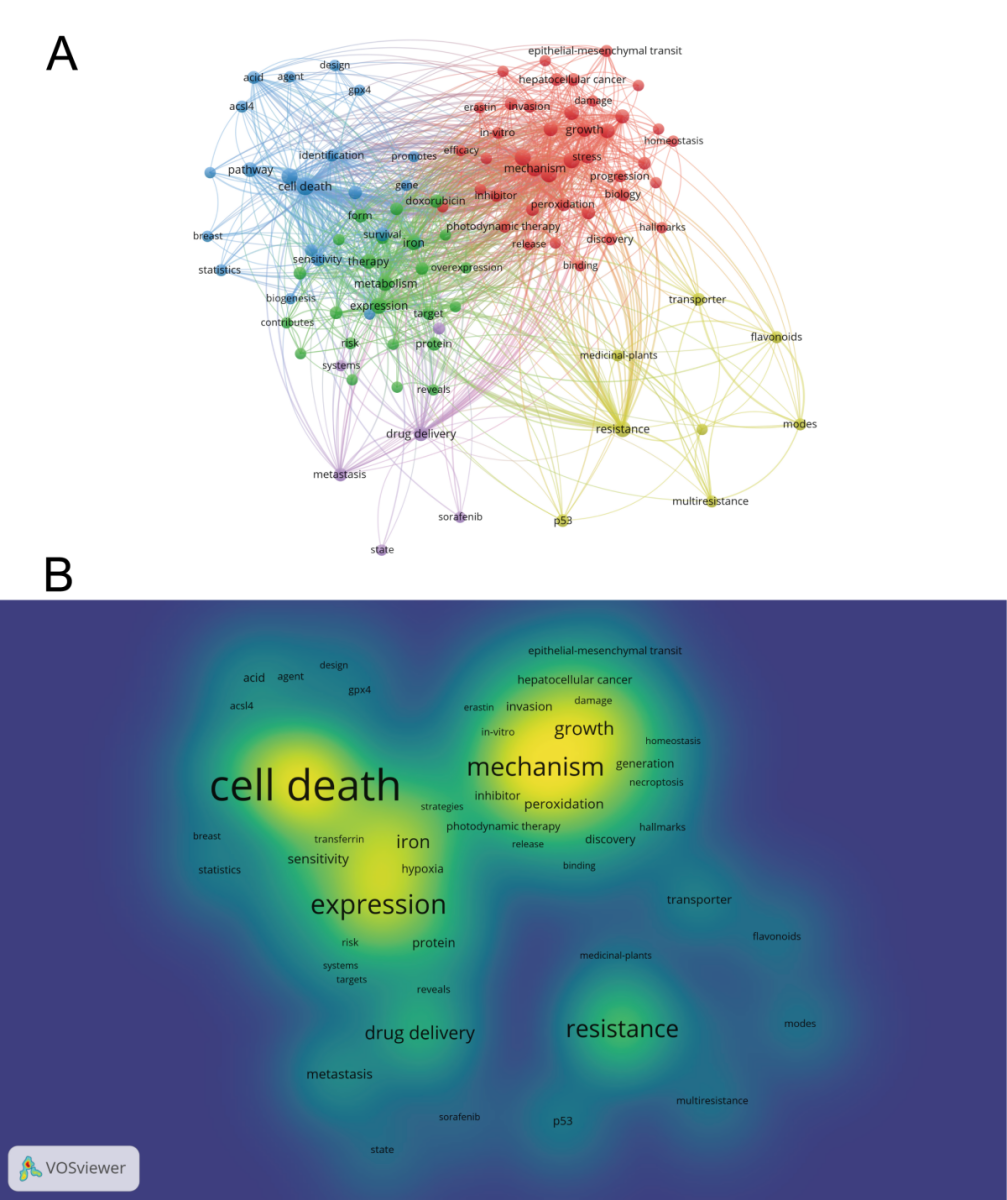

Supplement: Multimedia Appendix 7 [file ijmr_v14i1e66286_app7.png]
